# Supplementary material for: Stability and Antiproliferative Activity of Malvidin-Based Non-Oxonium Derivative (Oxovitisin A) Compared with Precursor Anthocyanins and Pyranoanthocyanins
Source: Molecules. 2022 Aug 7;27(15):5030. doi: 10.3390/molecules27155030 (PMC9370602; doi:10.3390/molecules27155030)
Supplement: Supplementary file 1 [file molecules-27-05030-s001.zip › Table S1.pdf]

Table S1. HPLC-DAD-MS characteristics of Mv3glc, Me-py, vitisin A and oxovitisin A.

Anthocyanins	RT/(min)	λmax/(nm)	MS/(m/z)	MS2/(m/z)	MS3/(m/z)
Mv3glc	11.21	538	493.04	331.02	-
Vitisin A	14.88	512	561.05	399.04	-
Me-py	21.09	478	530.99	369.03	-
Oxovitisin A	23.04	373	533.08	373.13	342.91
