# Supplementary material for: Stability and Antiproliferative Activity of Malvidin-Based Non-Oxonium Derivative (Oxovitisin A) Compared with Precursor Anthocyanins and Pyranoanthocyanins
Source: Molecules. 2022 Aug 7;27(15):5030. doi: 10.3390/molecules27155030 (PMC9370602; doi:10.3390/molecules27155030)
Supplement: Supplementary file 1 [file molecules-27-05030-s001.zip › Table S2.pdf]

Table S2. Reservation rates of Mv3glc, vitisin A, Me-py and oxovitisin A after heating at different temperatures and times.

reservation rate (%)	50 ℃		90 ℃	
	1 h	5 h	1 h	5 h
Mv3glc	97.36	83.04	73.79	29.52
Vitisin A	98.80	94.83	92.67	72.60
Me-py	99.28	96.39	96.63	86.30
Oxovitisin A	99.61	99.48	97.38	91.35
