# Supplementary material for: Stability and Antiproliferative Activity of Malvidin-Based Non-Oxonium Derivative (Oxovitisin A) Compared with Precursor Anthocyanins and Pyranoanthocyanins
Source: Molecules. 2022 Aug 7;27(15):5030. doi: 10.3390/molecules27155030 (PMC9370602; doi:10.3390/molecules27155030)
Supplement: Supplementary file 1 [file molecules-27-05030-s001.zip › Table S3.pdf]

Table S3. Thermal degradation parameters of Mv3glc, vitisin A, Me-py, and oxovitisin A.

**Anthocyanins**	**T(°C)**	k**(h^{-1})**	$T_{1/2}$**(h)**	**R^2**
Mv3glc	50	0.0406	17.07	0.970
	60	0.0643	10.78	0.960
	70	0.101	6.88	0.974
	80	0.138	5.04	0.971
	90	0.239	2.90	0.953
Vitisin A	50	0.0108	64.21	0.953
	60	0.0140	49.52	0.922
	70	0.0212	32.58	0.949
	80	0.0318	21.83	0.923
	90	0.0611	11.35	0.957
Me-py	50	7.737×10^{-3}	89.59	0.997
	60	0.0140	49.35	0.964
	70	0.0209	33.14	0.940
	80	0.0248	27.94	0.983
	90	0.0277	25.04	0.984
Oxovitisin A	50	5.261×10^{-4}	1317.52	0.750
	60	7.898×10^{-4}	877.62	0.921
	70	8.077×10^{-3}	85.82	0.974
	80	9.942×10^{-3}	69.71	0.858
	90	1.623×10^{-2}	42.71	0.972
