# Supplementary figures and images for: Stability and Antiproliferative Activity of Malvidin-Based Non-Oxonium Derivative (Oxovitisin A) Compared with Precursor Anthocyanins and Pyranoanthocyanins
Source: Molecules. 2022 Aug 7;27(15):5030. doi: 10.3390/molecules27155030 (PMC9370602; doi:10.3390/molecules27155030)

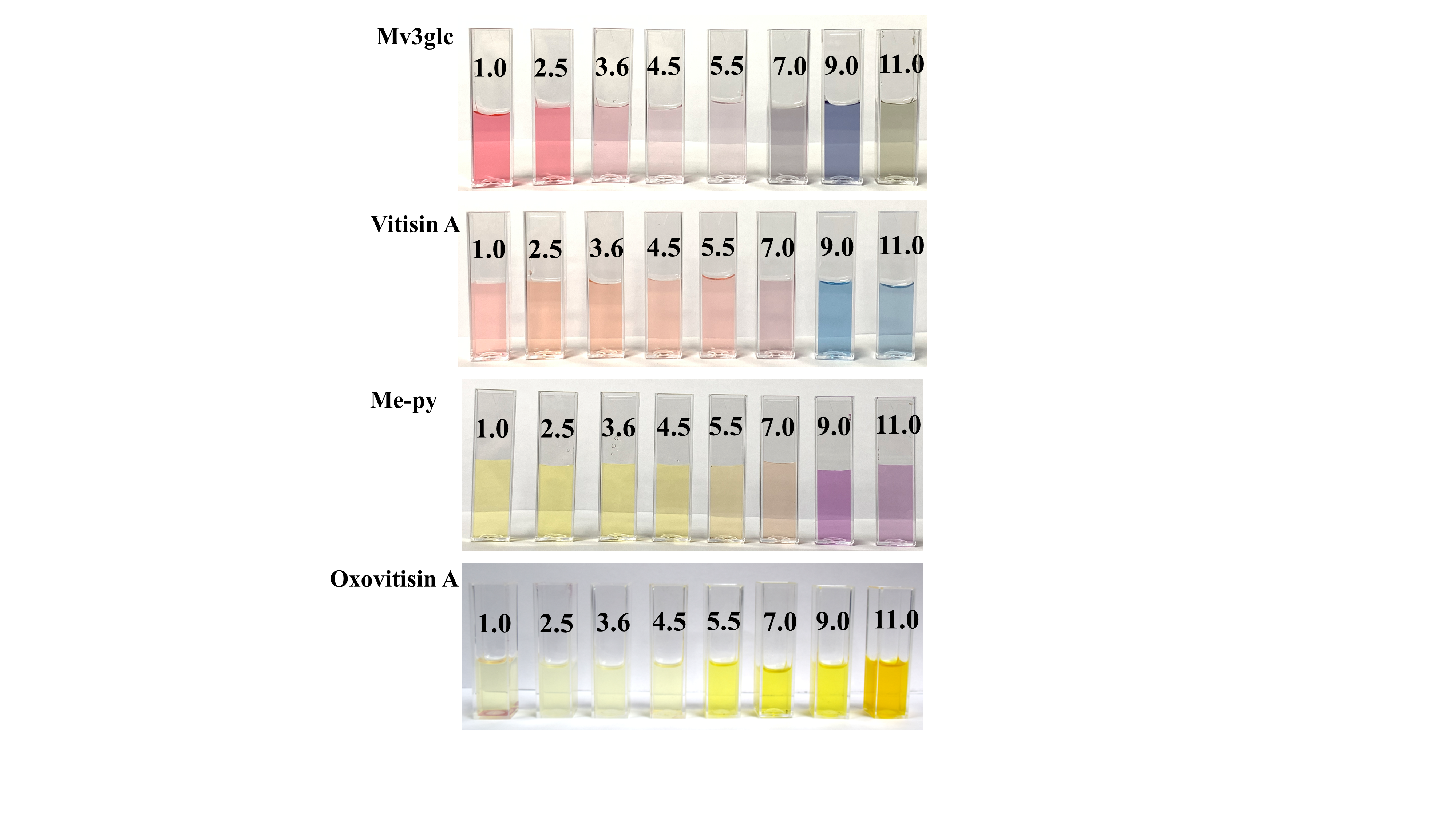

Supplement: Supplementary file 1 [file molecules-27-05030-s001.zip › Figure S1.tif]
